# The GSK3 hypothesis of Alzheimer's disease

**DOI:** 10.1111/j.1471-4159.2007.05194.x

**Published:** 2008-03

**Authors:** Claudie Hooper, Richard Killick, Simon Lovestone

**Affiliations:** King's College London, MRC Centre for Neurodegenerative Research, Institute of PsychiatryDe Crespigny Park, Denmark Hill, London, UK

**Keywords:** Wnt, insulin, tau, β-amyloid, memory, inflammation

## Abstract

Glycogen synthase kinase 3 (GSK3) is a constitutively active, proline-directed serine/threonine kinase that plays a part in a number of physiological processes ranging from glycogen metabolism to gene transcription. GSK3 also plays a pivotal and central role in the pathogenesis of both sporadic and familial forms of Alzheimer's disease (AD), an observation that has led us to coin the ‘GSK3 hypothesis of AD’. According to this hypothesis, over-activity of GSK3 accounts for memory impairment, tau hyper-phosphorylation, increased β-amyloid production and local plaque-associated microglial-mediated inflammatory responses; all of which are hallmark characteristics of AD. If our ‘GSK3 hypothesis of AD’ is substantiated and GSK3 is indeed a causal mediator of AD then inhibitors of GSK3 would provide a novel avenue for therapeutic intervention in this devastating disorder.

Alzheimer's disease (AD) is a neurodegenerative disorder defined by progressive memory loss and cognitive impairment and at the molecular level by the presence of neurofibrillary tangles (NFTs) and insoluble β-amyloid (Aβ) plaques (Hardy 2006) that are associated with activated microglia (Vehmas *et al.* 2003). NFTs are composed of hyper-phosphorylated forms of the microtubule-associated protein tau, whereas Aβ is derived from the proteolytic cleavage of β-amyloid precursor protein (APP). Early onset forms of Familial Alzheimer's disease (FAD) typically present before the age of 65 and have been linked to mutations in APP, presenilin-1 (PS-1) and presenilin-2 (PS-2). Mutations in these genes adversely affect APP processing and result in the increased production of insoluble Aβ and its deposition into plaques.

β-Amyloid precursor protein, presenilin and tau are undoubtedly pivotal to understanding the pathogenesis of AD and there are as yet no convincing refutations of the classical amyloid cascade hypothesis of AD, which postulates that, Aβ over-production leads to the pathogenic hyper-phosphorylation of tau resulting in the formation of neurofibrillary tangles (NFTs) and neurodegeneration. However, this hypothesis raises as many questions as it does answers. What regulates the processing of APP in sporadic AD as opposed to those very few families harboring APP or PS mutations? Which species of Aβ (Aβ42, Aβ*56, aggregated fibrillar Aβ or soluble oligomeric Aβ, intracellular Aβ or extracellular Aβ) is neurotoxic and how exactly does Aβ induce tau hyper-phosphorylation and does this lead to tau aggregation, neurotoxicity and loss of neuronal function? These are not idle questions, drug development in the AD field has concentrated very heavily on trying to alter APP processing, but developing alternative approaches is almost certainly going to be necessary for the effective treatment of this devastating and costly condition. We propose that glycogen synthase kinase-3 (GSK3) plays a leading role in the cascade of events that culminate in AD as this kinase is involved in the mechanisms underlying learning and memory, in the hyper-phosphorylation of tau, in the increased production of Aβ from APP and also in local cerebral inflammatory responses.

## GSK3 and cell signaling

There are two GSK3 genes from which GSK3α and GSK3β are derived. GSK3β also exists as longer splice variants (Mukai *et al.* 2002; Schaffer *et al.* 2003). GSK3α and GSK3β are ubiquitously expressed, constitutively active, proline-directed serine/threonine kinases involved in a variety of cellular processes including glycogen metabolism (Welsh and Proud 1993), gene transcription (Troussard *et al.* 1999), apoptosis (Turenne and Price 2001) and microtubule stability (Anderton *et al.* 2001; Brion *et al.* 2001). Insights from mouse models suggest that the GSK3 isoforms exhibit tissue specific physiological functions. GSK3β knock-out mice die *in utero* (Hoeflich *et al.* 2000), whereas GSK3α knock-out mice are viable and display improved glucose tolerance in response to glucose load and elevated hepatic glycogen storage and insulin sensitivity (MacAulay *et al.* 2007).

GSK3 activity is modulated by insulin and Wnt signaling, both pathways act in a negative regulatory manner. Many, but not all GSK3 substrates require pre-phosphorylation (priming) before phosphorylation by GSK3 can occur, so in both health and disease the activity of the priming kinase might limit GSK3 activity. Insulin signaling leads to the activation of PI3-kinase and subsequently the activation of acutely transforming retrovirus Akt8 in rodent T cell lymphoma (Akt otherwise known as protein kinase B), which in turn phosphorylates free cytoplasmic GSK3β and GSK3α at serine (Ser) residues 9 and 21, respectively (Saltiel and Kahn 2001; Lizcano and Alessi 2002). Regulatory serine phosphorylation results in the generation of an intra-molecular pseudo-substrate, which blocks part of the active site preventing the enzymatic activity of GSK3 towards primed substrates. This in turn leads to the de-phosphorylation of downstream substrates such as glycogen synthase and eukaryotic protein synthesis initiation factor-2B (eIF-2B), which elicits an increase in glycogen and protein synthesis. GSK3 can also be phosphorylated at Ser9/21 by p70 ribosomal S6 kinase-1 (S6K1) and by 90 kDa ribosomal protein S6 kinase (RSK), a downstream mediator of mitogen-activated protein kinase (MAPK) signaling (Doble and Woodgett 2003).

In addition to regulatory Ser phosphorylation, GSK3β and GSK3α activity can be regulated by tyrosine (Tyr) phosphorylation at residues 216 or 279, respectively. Under physiological conditions, GSK3 is phosphorylated at these sites and increases in Tyr phosphorylation augment GSK3 activity. However, regulation of GSK3 at Tyr 216/279 is perhaps less common than regulation at Ser9/21 (Bhat *et al.* 2000; Bijur and Jope 2001).

Wnt signaling regulates GSK3 activity by physically displacing complexed GSK3 from a number of regulatory binding partners consequently preventing the phosphorylation and degradation of β-catenin. In the absence of Wnt, the signaling pool of β-catenin is maintained at low levels through degradation (Dale 1998; Huelsken and Behrens 2002; Nusse 2005). β-catenin is targeted for ubiquitination by the β-transducin repeat containing protein (βTrCP) and is then degraded by the proteosome. β-catenin is phosphorylated by the serine/threonine kinases casein kinase 1 (CK1) and GSK3β. Phosphorylation of β-catenin occurs in a multi-protein complex (the destruction complex), comprising axin, adenomatous polyposis coli protein and diversin. Upon receipt of a Wnt signal, dishevelled prevents degradation of β-catenin through the recruitment of GSK3 binding protein (GBP)/Frat-1, which displaces GSK3β from the destruction complex. Stabilized β-catenin enters the nucleus and associates with T cell factor/lymphoid enhancer factor transcription factors, leading to the transcription of Wnt target genes, such as *cyclin D1, PPARδ and twin*. Recently, GSK3α in addition to GSK3β, has been shown to function in the destruction complex and the Wnt signaling pathway, suggesting that this enzyme is equally as important in Wnt biology as is GSK3β (Asuni *et al.* 2006; Doble *et al.* 2007).

## The role of GSK3 in AD

**Fig. 1:**
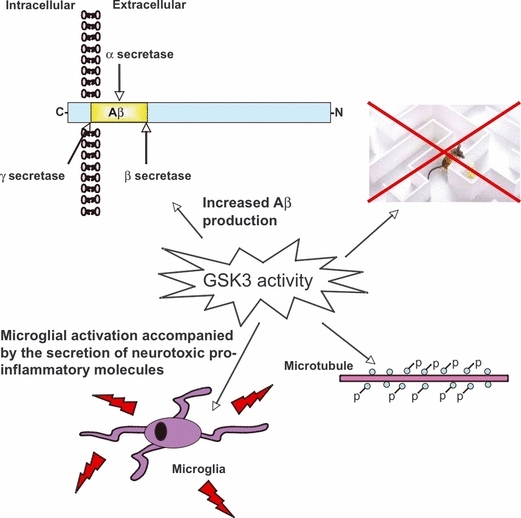
GSK3 and its role in AD. Over-activity of GSK3 caused either by aberrant Wnt or insulin signaling in sporadic AD cases or through familial mutations in PS or APP in FAD, might play an integral role in disease progression. GSK3 mediates the hyper-phosphorylation of tau, the increased production of Aβ from APP (via β and γ secretase-mediated cleavage) and results in impairments in learning and memory and could potentially heighten microglial-mediated inflammatory responses in the local vicinity of Aβ plaques.

The evidence that GSK3 plays a central role in AD and that its deregulation accounts for many of the pathological hallmarks of the disease in both sporadic and familial AD cases, has led us to formulate the ‘GSK3 hypothesis of AD.’ Evidence from our group and others suggests that GSK3 is intimately involved in the hyper-phosphorylation of tau, memory impairment, the increased production of Aβ and in inflammatory responses (Fig. 1). GSK3 also reduces acetylcholine synthesis, which is in accordance with the cholinergic deficit present in AD (Hoshi *et al.* 1996). Moreover, GSK3 is a key mediator of apoptosis (Turenne and Price 2001) and thereby might directly contribute to neuronal loss in AD.

Indeed, if GSK3 is central to AD pathogenesis then one would expect evidence for increased activity of this enzyme in AD. However, there is little such evidence, as it is technically difficult, if not impossible, to measure enzymatic activity in post-mortem brain tissue. Nevertheless, indirect evidence does support the role of GSK3 in disease. GSK3 has been shown to co-localize with dystrophic neurites and NFTs (Yamaguchi *et al.* 1996; Imahori and Uchida 1997; Pei *et al.* 1997). Active GSK3 appears in neurons with pre-tangle changes (Pei *et al.* 1999) and there is increased GSK3 activity in the frontal cortex in AD as evidenced by immuno-blotting for GSK3 phosphorylated at Tyr216 (Leroy *et al.* 2007). Furthermore, GSK3 expression is up-regulated in the hippocampus of AD patients (Blalock *et al.* 2004) and in post-synaptosomal supernatants derived from AD brain (Pei *et al.* 1997), although the latter study reports that there is not an increase in GSK3 enzymatic activity. GSK3 expression is also up-regulated in circulating peripheral lymphocytes in both AD and in mild cognitive impairment (Hye *et al.* 2005). It has recently been reported that a polymorphism in the GSK3 promoter is a risk factor for late onset AD (Mateo *et al.* 2006), which might account for alterations in GSK3 expression in disease. Collectively, these findings suggest that GSK3 activity might be increased in AD, through changes in its phosphorylation state as well as expression levels, although we acknowledge that direct evidence for this is still limited at present and some studies find no change in GSK3 activity (Pei *et al.* 1997) or reduced GSK3 activity (Swatton *et al.* 2004) in AD.

Genetic and epidemiological studies indicate that GSK3 is deregulated in AD through alterations in upstream Wnt and insulin signalling pathway intermediates. The low-density lipoprotein receptor related Protein 6 (LRP6), a co-receptor for Wnt signaling, has recently been identified as a risk gene for late onset AD in apolipoprotein E4-e4 negative individuals (De Ferrari *et al.* 2007), implicating aberrant Wnt signalling in AD pathology. In addition, an association of AD with diabetes and insulin resistance has been reported (Biessels and Kappelle 2005) and genetic studies find insulin signaling genes to be susceptibility loci for AD (Hamilton *et al.* 2007; Reiman *et al.* 2007).

## GSK3 and memory

Suppression of Wnt or PI3-kinase signaling impairs long term potentiation (LTP), the best characterized correlate of learning and memory (Sanna *et al.* 2002; Chen *et al.* 2006). GSK3 is negatively regulated by both the Wnt and insulin pathways and inhibition of GSK3β follows the induction of LTP in wild-type mice (Hooper *et al.* 2007; Peineau *et al.* 2007) suggesting that GSK3β might mediate the effects of insulin and Wnt on LTP. Accordingly, over-expression of GSK3β in mice prevents the induction of LTP (Hooper *et al.* 2007) and causes a decrease in spatial learning (Hernandez *et al.* 2002). Inhibitors of GSK3β have also been shown to block long-term depression (LTD) and GSK3β activity is enhanced during LTD (Peineau *et al.* 2007). Thus, it would appear that GSK3β is critical for the induction of memory formation, switching off LTD and allowing LTP to occur.

Many downstream substrates of GSK3 are involved in synaptic remodeling, which is a vital process required for the proper establishment of connections during memory formation. Adenomatous polyposis coli a synaptic scaffolding protein (Rubinfeld *et al.* 1996) and collapsin response mediator proteins (CRMPs) are known to influence growth cone dynamics and are hyper-phosphorylated in Aβ plaques (Cole *et al.* 2004). GSK3 also phosphorylates and inhibits cAMP responsive element-binding protein (Bullock and Habener 1998; Hansen *et al.* 2004), a universal modulator of memory. Moreover, GSK3 promotes actin and tubulin assembly (Koivisto *et al.* 2004), processes required for synaptic reorganization during memory formation. We propose, therefore, that GSK3 acts as a gate through which LTP and memory are established. Considering, GSK3 activity is increased in AD, memory failure in this disorder might be attributable to the inhibition of LTP with neuronal loss ensuing as the disease progresses.

## GSK3 and tau phosphorylation

Both GSK3β and GSK3α (Hanger *et al.* 1992; Ishiguro *et al.* 1992; Lovestone *et al.* 1994; Cho and Johnson 2003; Asuni *et al.* 2006) induce the hyper-phosphorylation of tau at both primed and non-primed phosphorylation sites, *in vitro* and in cell culture models of neurodegeneration, implicating GSK3 as an important tau-kinase possibly involved in the formation of NFTs *in vivo*. Consistent with this, GSK3β transgenic mice display tau hyper-phosphorylation and neurodegeneration (Lucas *et al.* 2001) and chronic lithium (GSK3 inhibitor) treatment prevents tau hyper-phosphorylation and NFT formation in double transgenic mice over-expressing GSK3β and tau (harboring a triple mutation associated with frontotemporal dementia and parkinsonism linked to chromosome 17), although reversal of pre-formed tangles was not observed (Engel *et al.* 2006). Similarly, double-transgenic *Drosophila* over-expressing tau and *shaggy*, the *Drosophila* form of GSK3, exhibit neuronal impairment and tau pathology (Mudher *et al.* 2004).

Insulin transiently increases tau phosphorylation in human neuroblastoma cells, but this is soon followed by a reduction in tau phosphorylation, which correlates with the activation and subsequent inhibition of GSK3 (Lesort *et al.* 1999). Dickkopf, a negative regulator of Wnt signaling also promotes tau phosphorylation and neurodegeneration through the activation of GSK3 (Caricasole *et al.* 2004) and Dickkopf is up-regulated in AD. Furthermore, PS-1 binds both GSK3β and tau, and mutant forms of PS-1, associated with FAD, bind GSK3β more effectively resulting in increased tau phosphorylation (Takashima *et al.* 1998). PS-1 has been shown to inactivate GSK3 through PI3-kinase/Akt signaling preventing tau phosphorylation and apoptosis. Interestingly, PS-1 FAD mutations inhibit PS-1-dependent PI3-kinase/Akt signaling, facilitating GSK3 activity and thus tau hyper-phosphorylation. This is one potential mechanism through which FAD mutations might lead to accelerated disease progression through secondary aberrations in GSK3 activity (Baki *et al.* 2004).

## GSK3 and Aβ production

GSK3α, but not GSK3β, has been shown to regulate APP cleavage resulting in the increased production of Aβ (Sun *et al.* 2002; Phiel *et al.* 2003). Exposure of neurones to Aβ increases GSK3β activity through the inhibition of PI3-kinase signalling and blockade of either GSK3β expression or activity prevents Aβ-induced neurodegeneration (Takashima *et al.* 1993, 1996; Alvarez *et al.* 1999). Although heightened GSK3 activity is not the primary cause of disease in this scenario, increased GSK3 activity would serve to augment Aβ production and in turn tau hyper-phosphorylation and neuronal degeneration in both FAD and sporadic cases, in line with the amyloid cascade hypothesis of AD.

Insulin signaling, possibly acting though the suppression of GSK3 activity, has also been shown to exert a beneficial effect on APP and amyloid processing. Insulin increases the expression of the Aβ-protease, insulin degrading enzyme (IDE) (Zhao *et al.* 2004) and enhances the secretion of sAPPα, which results from the non-amyloidogenic α-secretase-mediated cleavage of APP (Solano *et al.* 2000). We also note with great interest the finding that in people with AD, insulin given through the intranasal route favorably alters the Aβ40/42 ratio and improves cognition (Reger *et al.* 2007).

## GSK3 and inflammation

As well as being implicated in the core pathogenic events of AD – the increased formation of Aβ, tau hyper-phosphorylation and memory impairment – GSK3 might also play a role in other processes thought to impact on the amyloid cascade. Foremost amongst these are inflammatory processes. There is a growing body of evidence to suggest that inflammation plays an important role in AD. Microglia are known to accumulate around Aβ plaques in AD (Christie *et al.* 1996; Joshi and Crutcher 1998; Stalder *et al.* 1999) and there is increased expression of inflammatory mediators in AD brain tissue (McGeer and McGeer 1996). Moreover, Fc-dependent engagement of microglia is thought to be one mechanism through which Aβ immunotherapy operates. This treatment strategy has proven successful in improving cognitive function and reducing Aβ plaque burden in both animal models of AD and in human AD patients. However, phase II clinical trials were aborted as some patients developed symptoms of brain inflammation resembling encephalitis or meningitis following injection with Aβ (Robinson *et al.* 2003; Schenk *et al.* 2005). In the periphery, the regulation of GSK3 activity is critical for inflammatory cell differentiation, inflammatory cell migration and the secretion of pro-inflammatory cytokines (Woodgett and Ohashi 2005; Jope *et al.* 2007; Rodionova *et al.* 2007). However, little is known about the function of GSK3 in the cerebral inflammatory response, which is predominantly mediated by microglia. If, as we discuss above, GSK3 regulation is aberrant in AD brain, then this might impact upon the cerebral inflammatory response leading to the sustained secretion of neuro-toxic inflammatory mediators by microglia, in turn causing by-stander damage to neighboring neurons and contributing to neurodegenerative processes.

## Concluding remarks

There is then substantial data strongly implicating GSK3 in the pathogenesis of AD. GSK3 activity and/or protein levels are increased in afflicted individuals with AD and cell biological, epidemiological and genetic evidence points to an association between AD and pathways that regulate GSK3. We postulate these pathways augment GSK3 activity through secondary changes in both regulatory Ser and Tyr phosphorylation, mediated either directly or through alterations in the insulin and/or Wnt signaling cascades or indeed through other pathways that converge on GSK3.

In various cell culture, invertebrate and mammalian models of AD increasing GSK3 activity leads to the hyper-phosphorylation of tau, increased Aβ generation and deficits in learning and memory accompanied with neurodegeneration. Most importantly inhibiting GSK3 activity reverses some of the pathological effects of over-expression of mutated APP and tau in the best available models of AD (Noble *et al.* 2005; Rockenstein *et al.* 2007). Our ‘GSK3 hypothesis of AD’ integrates and extends the well established ‘amyloid cascade hypothesis of AD’ incorporating the known key molecular events and linking these with outcomes such as memory impairment and inflammation. If correct, then this hypothesis strongly implicates GSK3 inhibitors as a novel treatment strategy for AD.
